# Introduction to emerging systems

**DOI:** 10.1186/s13227-020-00153-y

**Published:** 2020-04-16

**Authors:** Michalis Averof, Chiara Sinigaglia

**Affiliations:** Institut de Génomique Fonctionnelle de Lyon (IGFL), CNRS and École and Normale Supérieure de Lyon, 32 Avenue Tony Garnier, 69007 Lyon, France

In 1929 August Krogh, a pioneer of comparative physiology, wrote: ‘For a large number of problems there will be some animal of choice, or a few such animals, on which it can be most conveniently studied’. Decades later Jacques Monod argued that anything found to be true of *E. coli* must also be true of elephants. These two ideas—that some species are particularly useful as experimental subjects, and that knowledge gained from these can apply broadly to other species—have deeply influenced molecular and developmental biology, and are the reasons we study model organisms.

Today, biological research is dominated by model organisms and it is widely assumed that most aspects of biology can be studied in just a few genetically tractable species—yeast, *Arabidopsis*, *Drosophila*, *C. elegans*, zebrafish and mice. Yet, in the early twentieth century Krogh’s statement was a call for expanding the scope of experimental models, not restricting it. In the same paragraph Krogh highlighted the relevance of exploring diverse organisms, by jokingly adding: ‘I have no doubt that there is quite a number of animals which are created for special physiological [research] purposes, but I am afraid that most of them are unknown to the men for whom they were created, and we must apply to the zoologists to find them and lay our hands on them’.

The aim of the *Emerging Systems* series, hosted by *EvoDevo*, is to help the research community lay their hands on lesser-explored organisms that widen our experimental and theoretical horizons.

There are at least two important motivations for wanting to broaden the scope of experimental research to new species. First, biological systems are the product of evolution and to understand the historical sequence and mechanisms of evolution requires comparisons among species that are separated by different evolutionary time spans. Since the early 1990s, interest in evolutionary developmental biology (evo-devo) has been a major driver for establishing new experimentally tractable species. Second, the well-established model organisms open a window on just a part of biology. What is true for *E. coli* is indeed often true for elephants, but there are aspects of the elephant that we cannot study in bacteria. The ability to regenerate the entire body must be probed in cnidarians and flatworms, the origins of multicellularity in volvocines, gene scrambling in ciliates, and so on. Some of the most exciting unresolved mysteries in biology lie outside the reach of classic model organisms. Conversely, a deeper understanding of seemingly ‘general’ concepts, such as the significance of germ layers or cell types, can be achieved only through a broader comparative approach.

The power of genetics in model systems is so seductive and the tools available so refined that it is sometimes tempting to look for proxies for these unsolved mysteries within the conventional models. The temptation to overstretch the use of model organisms reminds us of the well-known joke about the drunkard searching for his keys under a streetlamp because that is where there is light. When the keys lie away from the streetlamp, we must be prepared to probe darker places. In biology, that requires building or transferring new experimental approaches in new species.

*Emerging Systems* presents experimental organisms that broaden the scope of biological research, highlighting the importance of developing new experimental tools and approaches to explore diversity, the challenges in doing this, and the appeal of exploiting the unique features of each species. The articles include practical information on culture, tools and resources. We hope that the *Emerging Systems* series will inspire readers to think more broadly about the opportunities offered by different experimental systems, motivate them to explore new research directions, and perhaps to look at their current work from a different perspective.

